# Influence of diet on acute endocannabinoidome mediator levels post exercise in active women, a crossover randomized study

**DOI:** 10.1038/s41598-022-10757-0

**Published:** 2022-05-20

**Authors:** Fabiola Forteza, Isabelle Bourdeau-Julien, Guillaume Q. Nguyen, Fredy Alexander Guevara Agudelo, Gabrielle Rochefort, Lydiane Parent, Volatiana Rakotoarivelo, Perrine Feutry, Cyril Martin, Julie Perron, Benoît Lamarche, Nicolas Flamand, Alain Veilleux, François Billaut, Vincenzo Di Marzo, Frédéric Raymond

**Affiliations:** 1 Centre Nutrition, Santé et Société (NUTRISS), Institut sur la nutrition et les aliments fonctionnels (INAF), Quebec, Canada; 2 Centre de recherche de l’Institut universitaire de cardiologie et de pneumologie de Québec (IUCPQ), Quebec, Canada; 3 École de nutrition, Faculté des sciences de l’agriculture et de l’alimentation (FSAA), Université Laval, Quebec, Canada; 4 Département de médecine, Faculté de Médecine, Université Laval, Quebec, Canada; 5 Joint International Unit on Chemical and Biomolecular Research on the Microbiome and its Impact on Metabolic Health and Nutrition (UMI-MicroMeNu), Quebec, Canada; 6 Canada Research Excellence Chair in the Microbiome-Endocannabinoidome Axis in Metabolic Health (CERC-MEND), Quebec, Canada; 7 Département de kinésiologie, Faculté de médecine, Université Laval, Quebec, Canada

**Keywords:** Endocrinology, Metabolism, Nutrition, Lipidomics, Microbiome

## Abstract

The extended endocannabinoid system, also termed endocannabinoidome, participates in multiple metabolic functions in health and disease. Physical activity can both have an acute and chronic impact on endocannabinoid mediators, as does diet. In this crossover randomized controlled study, we investigated the influence of diet on the peripheral response to acute maximal aerobic exercise in a sample of active adult women (n = 7) with no underlying metabolic conditions. We compared the impact of 7-day standardized Mediterranean diet (MedDiet) and control diet inspired by Canadian macronutrient intake (CanDiet) on endocannabinoidome and short-chain fatty acid metabolites post maximal aerobic exercise. Overall, plasmatic endocannabinoids, their congeners and some polyunsaturated fatty acids increased significantly post maximal aerobic exercise upon cessation of exercise and recovered their initial values within 1 h after exercise. Most *N*-acylethanolamines and polyunsaturated fatty acids increased directly after exercise when the participants had consumed the MedDiet, but not when they had consumed the CanDiet. This impact was different for monoacylglycerol endocannabinoid congeners, which in most cases reacted similarly to acute exercise while on the MedDiet or the CanDiet. Fecal microbiota was only minimally affected by the diet in this cohort. This study demonstrates that endocannabinoidome mediators respond to acute maximal aerobic exercise in a way that is dependent on the diet consumed in the week prior to exercise.

## Introduction

The endocannabinoid system (ECS) is deeply involved in multiple metabolic functions in health and disease, as well as in the adaptation to acute/physiological or chronic/pathological deviations from a healthy and active lifestyle. The endocannabinoidome (eCBome) is defined as the expanded ECS^1, 2^ and is composed of a spectrum of endogenous bioactive lipids, i.e. the endocannabinoids anandamide (AEA) and 2-arachidonoyl-glycerol (2-AG), their congeners and their analogues, the receptors they bind to, and their biosynthetic and catabolic enzymes^3–5^. This system influences daily biological processes such as appetite/feeding, inflammation, stress, neurogenesis, motor activity/coordination, addiction, brain reward system^4, 6, 7^, depression/mood^1, 5, 8–10^ and circadian cycle^11^. The ECS is also a central contributor to exercise-related analgesia, sedation (post-exercise calm), anxiolysis and sense of well-being during running exertion^6, 8, 12^.

Beyond the simple action of exercising, the nature, intensity, frequency and duration of exercise can affect the plasmatic concentrations of eCBs and congeners^13–17^. It has been suggested that AEA levels are exercise-intensity dependent^13^ while 2-AG would be less responsive to exercise^14^, or showed inconsistent modulation between studies^18^. Additionally, although sex is one of the many factors affecting the eCBome and, as such, must be considered in studies among animals and humans^19^, only few studies were dedicated to investigate alterations in the female eCBome exclusively during physical activity^20, 21^. Among adult women, AEA, OEA and 2-AG peripheral levels evolve with acute aerobic exercise depending on the intensity, the degree of habitual exercise and, possibly, the fitness level through adaptive mechanism^22^.

On top of physical activity^6, 22^, the diet is a non-negligible environmental factor affecting eCB signalling, with nutrients and energy intake being correlated to the eCBome^1, 23, 24^. For instance, the increase of some eCBome mediators after a 3-day Mediterranean-inspired dietary intervention was associated to total intake of fatty acids, especially of the omega-3 type (n-3)^23^. Compared to western diets, the Mediterranean diet is characterized, among others, by increased docosahexaenoic acid (DHA) intake and associated with changes in gut microbiota structure and eCBome mediator levels^25^. Microbiota-mediated Mediterranean diet effects were proven to attenuate systemic inflammation^26^ and to protect from cardiometabolic complications^27^. The differences between the dietary regimens of the general population and athletes, in terms of energy, carbohydrates, fats and proteins, are notable and influence the compositional diversity of gut microbiota^1, 2, 28^. The impact of exercise and diet on the eCBome could thus be affected by the gut microbiota.

In this study, we focused on healthy active adult women, a demographic that is not extensively described in the literature, due to the common and premature discontinuation of sports among women^20, 21^. Our main objective was to determine the response of circulating eCBome mediators and short-chain fatty acids (SCFAs) to acute maximal aerobic exercise after 7 days of eating a Mediterranean-style diet (MedDiet) rich in n-3 fatty acids compared to a control diet reflecting average Canadian macronutrient intakes (CanDiet). We also verified the impact of the diet on gut microbiota composition to control, if required, for the potential relationship between the microbiome, diet, exercise and plasmatic eCBome mediators. We hypothesized that the dietary status of active women influences the change in eCBome mediators after an acute bout of maximal aerobic exercise, particularly NAEs^13, 14, 16, 22^, a class of lipid mediators that is emerging as being influenced by both the diet and gut microbiota composition^1, 2, 28^.

## Material and methods

### Ethics

This randomized, crossover and controlled feeding study was conducted at the Institute of Nutrition and Functional Foods (INAF) in Quebec City, Canada. Written informed consent was obtained at the initiation visit. The project was approved by Université Laval Ethics Committee (2019-005) and registered in the ClinicalTrials.gov registry (NCT04766528, 23/02/2021). All experiments were performed in accordance with relevant guidelines and regulations.

### Study participants

Candidates that were practising at least three hours of moderate to vigorous physical activity per week, including an hour of running for the last 3 months, and had no signs of cardiometabolic and enteric disorders were admissible. Women who were pregnant, breastfeeding and/or in menopause, smoking, exceeding seven alcoholic beverages per week, had chronic disease, had taken antibiotics in the last 3 months^29^ or experienced an important weight change (± 5 kg) in the past 6 months could not take part in the study. Physical activity readiness based on cardiovascular health and musculoskeletal function was assessed with the Canadian Society for exercise physiology questionnaires and part of the eligibility conditions^30^. Twelve active women of 19 to 32 years old were recruited by a research assistant in September 2019 and seven participants completed the study successfully (Table 1). Participants were removed from the study for the following reasons: antibiotics in the week before study (2), antibiotics during study (1), no longer available (1), important delays between aerobic exercise test and blood sampling (1).

**Table 1:** Initial characteristics of subjects (n = 7).

	Mean ± SD	Range
Age (yr)	25 ± 5	19–32
BMI (kg/m^2^)	22.52 ± 1.57	19.50–24.49
Waist circumference (cm)	78.32 ± 5.54	68.50–85.67
Energetic need (kcal/jr)	2306.1 ± 310.8	1822.0–2853.0
Heart rate at rest (bpm)	61 ± 12	48–83
Physical activity (min/week)	473 ± 162	225–720

### Study design

**Figure 1:**
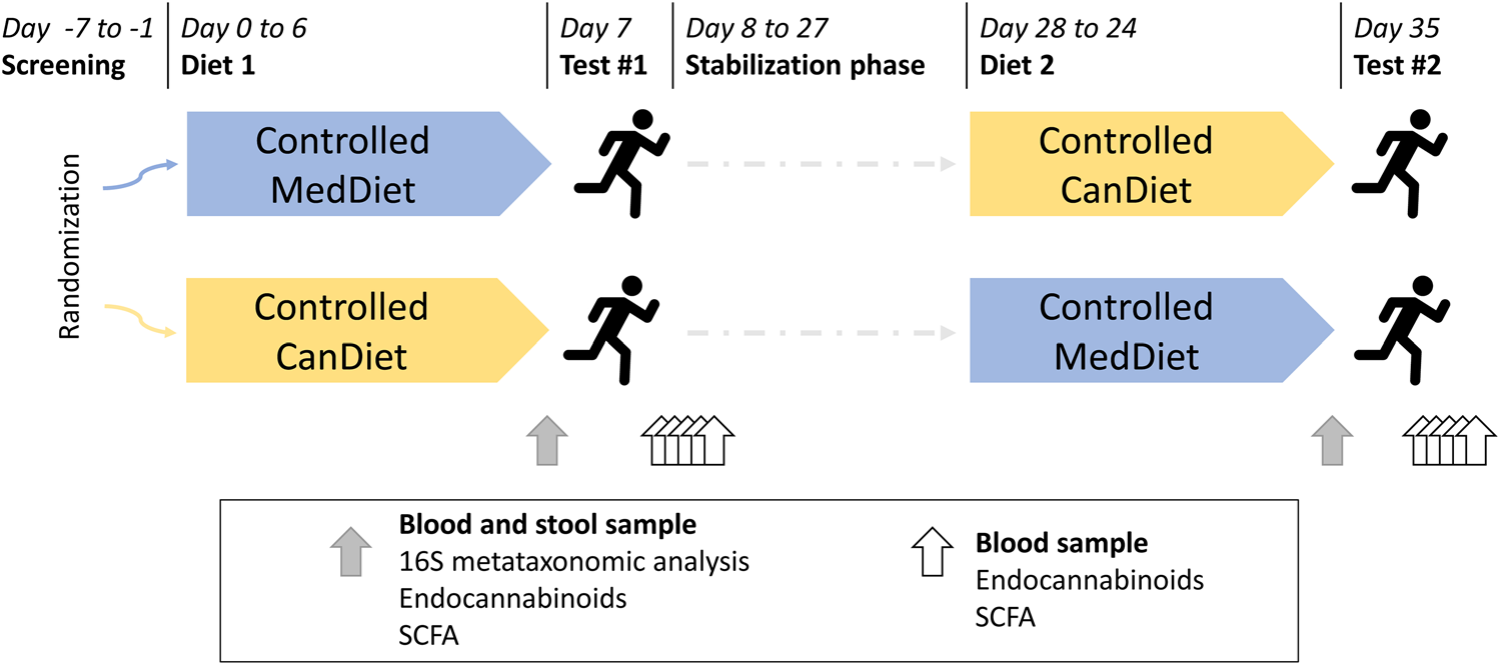
Schematic representation of the randomized crossover format of the study and the process from the start to the end including the periods of restricted diet, aerobic tests, samples taken and analyses performed.

Design of the study is summarized in Fig. 1. Participants were randomized (simple randomization) to two treatment sequences (MedDiet first followed by CanDiet, or vice versa) using a computer-generated list. Final sequence assignment was 4 CanDiet-MedDiet and 3 MedDiet-CanDiet. Thus, analyses include 7 participants for which response to CanDiet and MedDiet are compared. Participants had to come to INAF for an initiation visit. They also had to complete web-based self-administered questionnaires^31–34^. Afterwards, they received a 7-day MedDiet or 7-day CanDiet. All food and caloric beverages were provided to participants in the form of three meals based on a 7-day menu. After the first dietary intervention, participants underwent a 3-week stabilization period returning to their usual diet. In addition, this duration was selected to increase the probability that participants performed the second dietary intervention in the same menstrual cycle phase as the first dietary intervention. This 3-week stabilization period was coherent with the 28 days average cycle length of participants and use of oral contraceptives in 5 out of 7 participants. Thereafter, participants received 7-day of the diet they did not receive first. Acute maximal aerobic tests were performed at the end of every dietary intervention. The total duration of the study was 35 days (October 10 to November 14, 2019). Throughout the study, participants were asked to maintain their usual level of physical activity.

### Initiation visit

At the initiation visit, participants were 12 h-fasted, 48 h alcohol-abstinent and had followed their usual physical activity load. Blood samples were collected to evaluate lipid, glycemic and hormonal profiles to confirm participants did not have pathological conditions (data not shown). Weight was measured to the nearest 0.1 kg on a calibrated balance and height was measured to the nearest millimetre with a stadiometer. Body mass index (BMI) was then calculated (kg/m^2^). Waist circumference measured twice to the nearest millimetre by a standardized procedure at the iliac crest. Throughout the dietary intervention, the body mass was measured regularly and caloric needs were respected to ensure that the weight remained stable^35–37^.

### Dietary interventions

The MedDiet provided to the participants was formulated based on characteristics of the traditional Mediterranean eating pattern, including high levels of fibres, micronutrients and plant-based proteins (Table 2). The diet was rich in monounsaturated fatty acids (MUFAs) and n-3 polyunsaturated fatty acids (PUFAs). The control diet (CanDiet) was a Western-style diet designed to reflect current Canadian macronutrient intakes while not causing short-term nutritional deficiencies. The CanDiet was low in fibre and had a high content of saturated fatty acids (SFAs) and simple sugars. Both experimental diets were based on a 7-day menu developed using the Nutrition Data System for Research software (version 2011, Nutrition Coordinating Center, University of Minnesota) and used in previous studies by our group^38^. Participants were told to consume all and only what was provided. As an exception, they were allowed to consume caffeine-containing drinks with a limit two cups/day. Food consumption was validated using a checklist provided to all participants to identify the prescribed foods that had truly been consumed when eating the diets. This list also provided space to indicate unlisted food items that had been consumed in addition to the formulated diets if applicable.

**Table 2:** Macronutrients and micronutrients composition of the CanDiet and the MedDiet.

	CanDiet	MedDiet
Energy (KCAL)	2485	2502
HEI score	57.2	86.4
Carbohydrates (%E)	51.77	50.64
Proteins (%E)	16.88	16.68
Lipids (%E)	31.16	28.62
Alcohol (%E)	0.08	4.40
SFA (%E)	13.37	5.76
Monounsaturated fatty acids (MUFA) (%E)	10.41	15.66
PUFA (%E)	4.81	4.97
SFA 16:0 (palmitic acid) (G)	17.84	11.28
MUFA 18:1 (oleic acid) (G)	27.35	43.02
PUFA 20:4 (arachidonic acid [AA]) (G)	0.13	0.15
PUFA 20:5 (eicosapentaenoic acid [EPA]) (G)	0.01	0.12
PUFA 22:5 (docosapentaenoic acid [DPA]) (G)	0.01	0.04
PUFA 22:6 (docosahexaenoic acid [DHA]) (G)	0.06	0.27
PUFA/SFA ratio	0.36	0.88
Total dietary fiber (G)	21.60	48.57
Sodium (MG)	3626.2	2738.7
Calcium (MG)	1023.0	1242.1
Vitamin D (MCG)	5.8	6.3

### Acute maximal aerobic exercise test

The acute maximal aerobic exercise test involved the completion of two maximal aerobic exercise tests of 10 to 20 min on an indoor track. We used the progressive and continuous aerobic test protocol VAM-EVAL to directly measure aerobic maximal speed and estimate the maximal O_2_ consumption (VO_2_max, ml/min/kg), i.e. aerobic capacity, of the participants^39^. The participants started the test at a pace of 7 km/hour on a marker and ran through the next marker at a 20 m distance. The pace was prescribed by audio recording, increasing by 0.5 km/h every minute. The participants were asked to continue running until volitional exhaustion or were stopped by the experimenter when they missed 2 successive markers. We used CR10 Borg Scale to ensure they had reached a high physical intensity of 8/10 at least (Borg, 1982). Participants executed the two tests at the same time of the day at the two visits to limit intraindividual lipid mediator fluctuations due to circadian fluctuations^11^.

#### Blood and fecal sampling

Two certified nurses supervised blood sampling as well as participants’ clinical wellbeing in collaboration with the kinesiologists. One brachial blood extraction pretest (T_pre_) was made. With the help of a catheter, the first post-exercise sampling was taken at the nearest time point at the end of the aerobic challenge, corresponding to T_0_ and the others 15 (T_15_), 30 (T_30_), 45 (T_45_) and 60 (T_60_) min later. The fecal collection was carried out the day prior to each aerobic maximal test and immediately frozen by the participant.

### Plasma endocannabinoids, congeners and fatty acids quantification

Levels of NAEs, MAGs and PUFAs in plasma samples (300 μL) were measured using high-performance liquid chromatography coupled to tandem mass spectrometry (LC–MS/MS)^40^. It allowed the quantification of NAEs including anandamide (AEA), palmitoyl-ethanolamine (PEA), oleoyl-ethanolamine (OEA), *N*­linoleoyl-ethanolamine (LEA), *N*­stearoyl-ethanolamine (SEA), *N*­docosapentaenoyl‑ethanolamine (DPEA), *N*­eicosapentaenoyl-ethanolamine (EPEA) and *N*­docosahexaenoyl‑ethanolamine (DHEA), as well as MAGs including 1/2-arachidonoyl-glycerol (AG), 1/2-palmitoyl-glycerol (PG), 1/2-oleoyl-glycerol (OG), 1/2-linoleoyl-glycerol (LG), 1/2-eicosapentaenoyl-glycerol (EPG), 1/2-docosaepentaenoic-glycerol (DPG) and 1/2-docosahexaenoyl-glycerol (DHG). Monoacylglycerol isomers at positions 1 and 2 can be differentiated using this method. However, given their rapid interconversion and the preferential esterification of PUFA on the sn-2 position of phospholipids, MAGs quantified in this study were summed and identified as 2-MAGs. Unesterified PUFAs, including arachidonic acid (AA), docosahexaenoic acid (DHA), docosapentaenoic acid (DPA) and eicosapentaenoic acid (EPA), were also measured.

### Plasma short chain fatty acids quantification

Plasma samples were collected and kept at − 80 °C until SCFA extraction and measurement by gas chromatography. After 2 min centrifugation at 18,000*g* at 4 °C, 2 aliquots of plasma were acidified with H_3_PO_4_ 10% to obtain a pH around 2, and deproteinized with 5% v/v of sulfosalicylic acid 500 mg/mL. Samples were then centrifuged 5 min at 18,000*g* at 4 °C, and supernatants were transferred to a glass vial. An equal volume of methyl tert-butyl ether was added to extract SCFAs by vortexing 2 min. Extracts were centrifuged at 5500*g* for 10 min at 4 °C to separate organic and aqueous phases. SCFA analysis was performed on a GC-FID system (Shimadzu), consisting of a GC 2010 Plus gas chromatograph equipped with an AOC-20s auto-sampler, an AOC-20i auto-injector and a flame ionization detector. The system was controlled by GC solution software. Two microlitres of organic phase were injected in a split mode into a Nukol capillary GC column (30 m × 0.25 mm id, 0.25 µM film thickness, Supelco analytical) and hydrogen was used as carrier gas. The injector and detector were set at 250 °C. The oven temperature was initially programmed at 60 °C, then increased to 200 °C at 12 °C/min, which was held 7 min. SCFA were quantified using a 5-point calibration curve prepared with a mix of standards (acetic acid, propionic acid, butyric acid, isobutyric acid and valeric acid) extracted following the same procedure as plasma samples.

### Stool collection and metataxonomic analysis

Stool microbial DNA was extracted using the QIAamp DNA Stool Mini Kit (QIAGEN, CA, USA)^23^. Amplification of the 16S ribosomal RNA region V3-V4 was performed using the forward primer S-D-Bact-0341-b-S-17 (5=CCTACGGGNGGCWGCAG) and reverse primer S-D-Bact-0785-a-A-21 (5=GACTACHVGGGTATCTAATCC)^41^ concatenated with the Illumina adapters used for the Nextera library prep kits (Illumina, CA, USA). Briefly, each library was amplified in a total volume of 20 µL that contained 0.25 µM of each primer, 10 µL of 2X Phusion High-Fidelity PCR Master Mix (Thermo Scientific, Vilnius, Lithuania) and 1 µL of microbial DNA. The PCR started with an initial denaturation at 98 °C for 2 min followed by 35 cycles of denaturation at 98 °C for 10 s, annealing at 55 °C for 30 s, extension at 72 °C for 30 s followed by a final extension step at 72 °C for 2 min. PCR reactions were diluted to a fifth and used as a template for barcoding PCR. Second PCR was done in a total volume of 10 µL with 0.5 µL of each dual-index primer from the Nextera XT DNA Library Prep Kit (Illumina, CA, USA), 1 µL of the diluted cDNA template, 3 µL of UltraPure DNase/RNase-Free Distilled Water and 5 µL of 2X Phusion High-Fidelity PCR Master Mix (Thermo Scientific, Mississauga, Canada). The second cycle used the same cycling parameters, but with eight cycles. Barcoded Amplicons were then pooled in equimolar concentration and purified using AMPure XP beads (Brea, CA, USA). Libraries were sequenced on the Illumina Miseq.

Raw sequences were demultiplexed following the Illumina default parameters. Raw sequencing reads are available in SRA (PRJNA758983). Sequence assignation to amplicon sequence variants (ASV) was done in R version 3.1.2 using Dada2 package version 1.5.0^42^. Primer sequences were removed using cutadapt 3.2. Sequences were trimmed at 270 pb for forward reads and 210 pb for reverse reads. Amplicon error learning for the forward and reverse reads was performed on 100 M nucleotides using the DADA2 algorithm with default parameters. Denoised output reads were generated by dereplication and all reads with any mismatches were removed. Forward and reverse reads were merged, and chimera sequences were removed with the uchime_denovo function from vsearch package with a minimum difference set at 2. Purged ASV sequences with more than 4 read counts were kept. ASV assignation to bacterial taxa was performed by alignment based on the silva database version 138^43^. Finally, samples have been rarefied at 13 827 reads per sample. Statistical analyses were only performed on OTU that were at more than 1% in at least one sample.

### Statistical methods

All figures and statistical analyses were generated using R studio software. Mixed linear-effect models (LME) including random individual effect was used to identify metabolites influenced by diet, the time of sampling and the interaction between these two factors. The primary objective was to determine if plasma lipid mediators were differentially modulated after exercise following different dietary interventions. Plasma lipid concentrations have been normalized using ranked values fitted into LME (~ diet * time) and significance has been tested by analysis of variance (ANOVA) with random effects nested within participants. A p-value lower than 0.05 was considered significant. The secondary objective was to determine if microbiome composition was differentially modulated following dietary interventions. A fixed effect linear model (~ diet) was used to test the influence of diet on the microbiota families. A p-value lower than 0.05 was considered significant. Principal component analysis (PCA) and multiple factor analysis (MFA) were made using the ‘FactoMineR' package. PCA and MFA plots were made with fviz_pca and fviz_mfa functions of the 'factoextra' package. Metabolite response plots and microbiota families dot plots were drawn with the ‘ggplot2’ package. Permutational multivariate analysis of variance (PERMANOVA) has been made using adonis function of the ‘vegan’ package with a number of permutations of 100,000. Hierarchical clustering of microbiome samples were based on Canberra distance. Barplots were drawn with barplot2 function from the ‘gplots’ package.

## Results

Active female participants were recruited for this randomized crossover study to assess the impact of diet on eCBome mediators response post maximal aerobic exercise. Overall, participants had BMI and waist circumference averages representing a healthy body weight (Table 1). The mean estimated VO_2_max of participants was categorized as “sportive” for 20–29 year old individuals (Table 3)^44^. Likewise, the average heart rate at 5-min post-test was slightly below the 101–102 bpm of healthy subjects after a short treadmill exercise test reported in previous studies^45, 46^. The estimated VO_2_ max and the max heart rate were similar in both tests thus confirming that the intensity and effort were similar between diet interventions.

**Table 3:** Aerobic measurements after CanDiet and MedDiet.

	CanDiet ± SD (n = 7)	Range	MedDiet ± SD (n = 7)	Range
Mean exercise time (minutes)	10.9 ± 5.1	4–20	11.4 ± 5.1	4–20
Mean exercise HR (bpm)	164 ± 8	154–177	168 ± 9	158–181
Estimated max HR (bpm)	195 ± 5	188–201	195 ± 5	188–201
Maximal HR (bpm)	191 ± 6	182–201	190 ± 5	182–200
% of estimated max HR achieved	98 ± 2.7	95–102	98 ± 2.4	95–101
HR recovery 5 min post-test (%)	− 50.5 ± 3.5	− 57.29/− 46.56	− 51.9 ± 4.0	− 57.89/− 45.21
Estimated mean VO_2_ max (ml/min/kg)	43.5 ± 9.0	31.5–59.5	44.5 ± 9.0	31.5- 59.5
Borg rating of perceived exertion (RPE)	8.9 ± 0.7	8–10	9.3 ± 0.5	9–10

### Plasma N-acyl-ethanolamines post-exercise response differs between diets

**Figure 2:**
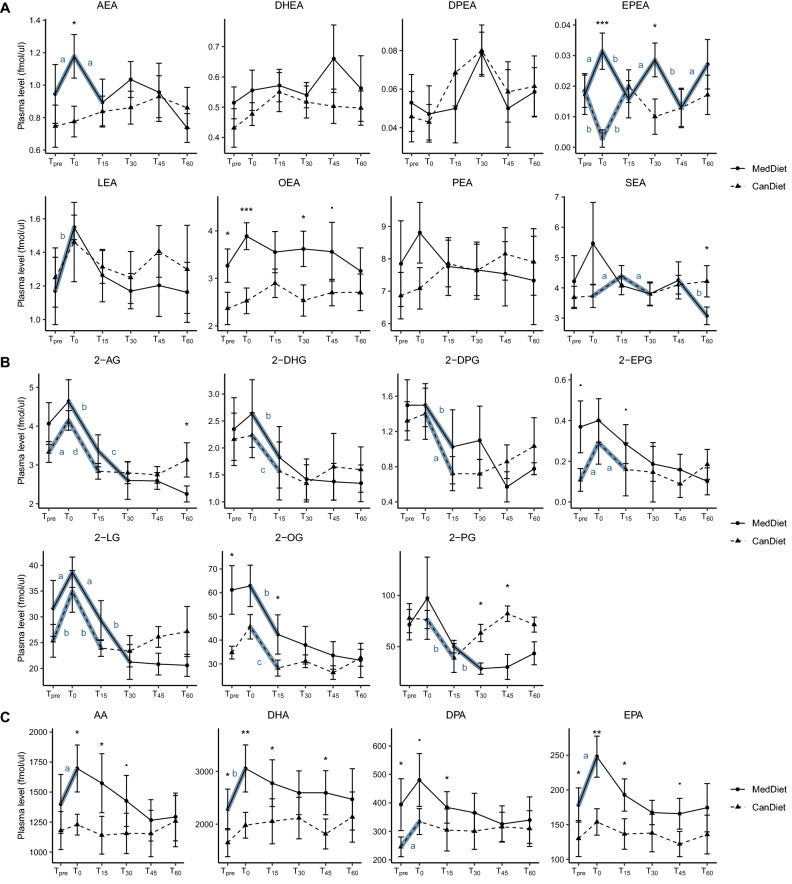
Response curves of eCBome mediators and precursors (fmol/μl) to maximal aerobic test according to the diet. Panels show results for (**A**) *N*-acylethanolamines, (**B**) monoacylglycerols and (**C**) polyunsaturated fatty acids. The graph represents the mean with the standard error of the plasmatic concentrations of these lipids at each time point for the two diets. T_pre_ corresponds to the initial concentration before the aerobic test, T_0_ corresponds to the immediate end of the aerobic test and T_15_, T_30_, T_45_ and T_60_ correspond to the recovery phase. Lipid concentrations have been normalized using ranked values fitted into mixed linear-effect models (LME) and differences between diets and times have been tested by analysis of variance (ANOVA). For difference between diets, significance was set at p < 0.1 (.), p < 0.05 (*), p < 0.01 (**) and p < 0.001 (***). For difference between times, indicated by bold blue line, significance was set at p < 0.1 (a), p < 0.05 (b), p < 0.01 (c), p < 0.001 (d). N = 7 per diet. Names of the molecules are anandamide (AEA), *N*-palmitoylethanolamine (PEA), *N*-oleoylethanolamine (OEA), *N*­linoleoylethanolamine (LEA), *N*-stearoylethanolamine (SEA), *N*­docosapentaenoylethanolamine (DPEA), *N*­eicosapentaenoylethanolamine (EPEA), *N*­docosahexaenoyl‑ethanolamine (DHEA), 1- plus 2-arachidonoyl-glycerol (2-AG), 1- plus 2-palmitoyl-glycerol (2-PG), 1- plus 2-oleoyl-glycerol (2-OG), linoleoyl-glycerol (2-LG), 1- plus 2-eicosapentaenoyl-glycerol (2-EPG), 1- plus 2-docosaepentaenoic-glycerol (2-DPG), 1- plus 2-docosahexaenoyl-glycerol (2-DHG), arachidonic acid (AA), docosahexaenoic acid (DHA), docosapentaenoic acid (DPA) and eicosapentaenoic acid (EPA).

Plasma *N*-acyl-ethanolamines (NAEs) showed post-exercise profiles dependent on the diet (Fig. 2A). Before exercise, only OEA was significantly increased by the MedDiet compared to CanDiet. This mediator remained significantly higher with MedDiet compared to CanDiet at several time points. DPEA, DHEA and LEA had similar response curves between the two diets. Statistically significant increases at T_0_, i.e. immediately after exercise, were observed following the MedDiet for AEA and LEA. No significant increase at T_0_ was observed for OEA, PEA and SEA following the MedDiet and for all NAEs following the CanDiet.

### Plasma 2-monoacylglycerol post exercise profiles were similar between diets

In general, plasmatic 2-monoacylglycerols (2-MAGs) reacted similarly to acute maximal aerobic exercise after the MedDiet and CanDiet (Fig. 2B). However, before exercise, 2-OG and 2-EPG were significantly higher with the MedDiet than with the CanDiet at T_pre_. Peaks at T_0_ after exercise were observed for 2-AG and 2-LG in both diets. Most 2-MAG concentrations decreased significantly as soon as 15 min after exercise. After CanDiet, most 2-MAGs, excluding 2-DHG and 2-DPG, had similar concentrations at T_pre_ and T_60_, which suggests a return to basal levels after exercise. After the MedDiet, 2-MAGs had lower concentrations after 60 min recovery (T_60_) than their initial value (T_pre_).

### Plasma PUFA post exercise response was diet dependent

Plasma concentrations of PUFAs before acute maximal aerobic exercise were significantly higher with the MedDiet compared to the CanDiet, except for arachidonic acid (AA) (Fig. 2C). For all time points, both before and after exercise, average PUFA levels were higher with the MedDiet than the CanDiet. Plasma PUFA levels peaked at T_0_ post exercise, remained high 15 min and returned to their initial levels at T_60_ only when participants consumed the MedDiet. When participants consumed the CanDiet, plasma fatty acid composition remained stable through time and only marginal changes in relation to exercise were observed for the n-3 PUFAs.

### Plasmatic SCFA post exercise response was similar between diets

**Figure 3:**
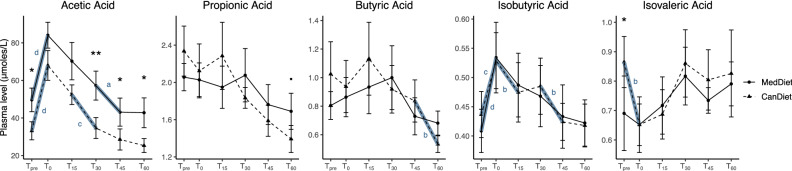
Response curves of SCFA to maximal aerobic test according to the diet. The graph represents the means with the standard errors of the plasmatic SCFA concentrations at each time point for the two diets. T_pre_ corresponds to the initial concentration before the aerobic test, T_0_ corresponds to the immediate end of the aerobic test and T_15_, T_30_, T_45_ and T_60_ correspond to the recovery phase. SCFA concentrations have been normalized using ranked values fitted into mixed linear-effect models (LME) and differences between diets and times have been tested by analysis of variance (ANOVA). For difference between diets, significance was set at p < 0.1 (.), p < 0.05 (*), p < 0.01 (**) and p < 0.001 (***). For difference between times, indicated by bold blue line, significance was set at p < 0.1 (a), p < 0.05 (b), p < 0.01 (c), p < 0.001 (d). N = 7 per diet.

We quantified plasma levels of SCFAs in response to acute maximal aerobic exercise and diets (Fig. 3). Acetic acid had significantly higher concentration with the MedDiet compared to the CanDiet at T_pre_. On the contrary, isovaleric acid concentration was significantly higher with the CanDiet compared to the MedDiet before exercise, but its levels dropped after exercise at T_0_. The acetic acid curve had a similar shape between the two dietary interventions, with concentrations that remained significantly superior in the MedDiet compared with the CanDiet throughout the protocol. The isobutyric acid response was nearly identical between the two diets, with a strong peak at T_0_ with MedDiet and CanDiet and a gradual return to initial level at later time points. Other SCFAs presented modest reactivity to acute maximal aerobic exercise in either MedDiet or CanDiet.

### Overview of eCBome and SCFA post exercise response between diets

**Figure 4:**
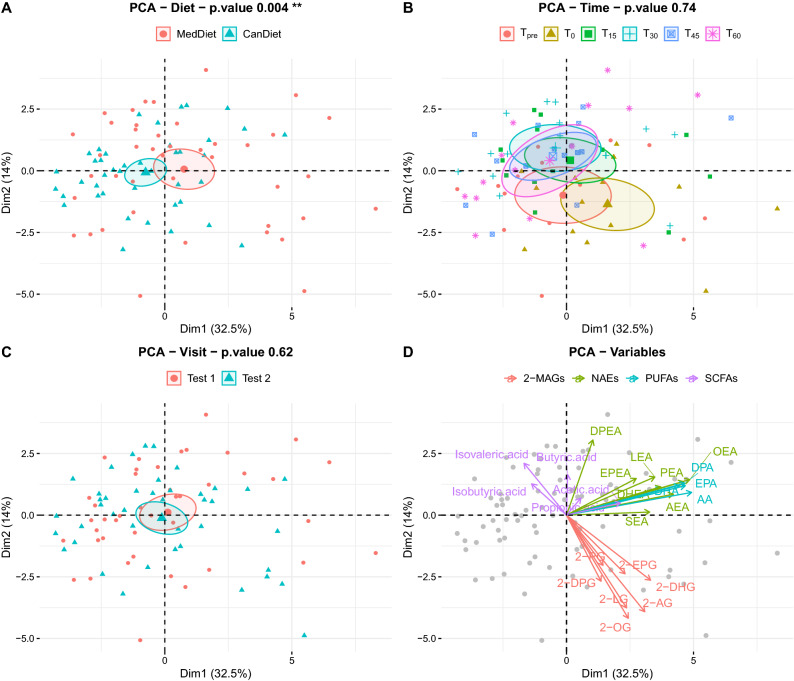
Representation of plasmatic lipid mediators and SCFA profiles shows an effect of diet and time post-exercise on the endocannabinoidome. Principal component analysis (PCA) of plasmatic profiles of free n-3 PUFA, endocannabinoids and endocannabinoid-like mediators and SCFA, where ellipses show how these metabolites differentiate (**A**) diet, (**B**) time points and (**C**) visits. (**D**) Loading plot shows the influence of plasmatic lipids on the PCA plot. P-values on PCA were obtained by permutational multivariate analysis of variance (PERMANOVA).

We used a principal component analysis (PCA) to represent all plasmatic mediators together, including NAEs, MAGs, PUFAs and SCFAs (Fig. 4). The difference between MedDiet and CanDiet was significant (Fig. 4A), supporting the concept that the diet influences the response to exercise of eCBome mediators and/or bioactive gut microbiota metabolites such as SCFA (p = 0.004**, PERMANOVA). The PCA showed differences between MedDiet and CanDiet in plasma metabolite levels at baseline (T_pre_), immediately after exercise (T_0_) and at later recovery time points (Fig. 4B). However, we observed an overlap of the samples at 15, 30, 45 and 60 min after the exercise. The order in which participants received the diets had no effect on the lipid profile (Fig. 4C). Variable contribution to PCA dimensions indicates associations between NAE and PUFA levels with diets (Fig. 4D). 2-MAGs and SCFAs were generally associated with time.

### Standardized diets had limited impact on active women fecal microbiota

**Figure 5:**
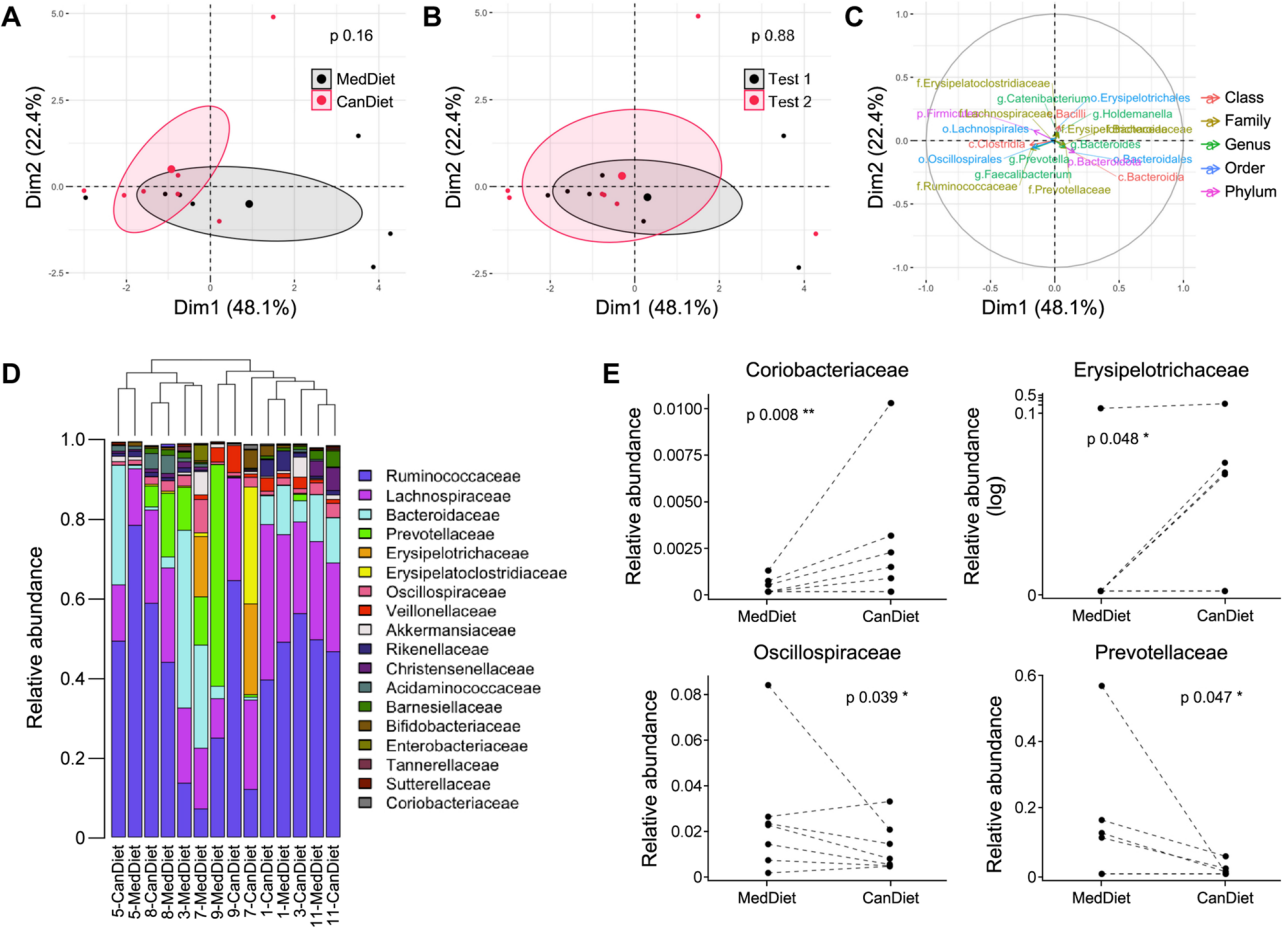
Multiple factor analysis (MFA) of the gut microbiota profile. Ellipses show the effect of (**A**) diet and (**B**) visit on individuals at different time points. (**C**) Loading plot shows the influence of the top 30 microbiota taxa with the highest contribution on the MFA plot. P-values on MFA were obtained by permutational multivariate analysis of variance (PERMANOVA). (**D**) Barplot representing the relative abundance of the 18 more abundant gut microbiota’s families. Dendrogram showing hierarchical clustering on Canberra distance between samples is printed above the barplot and determines the sample order. (**E**) Relative abundance of bacterial families significantly different between diet. Relative abundance of microbiota taxa has been normalized using ranked values fitted into mixed linear-effect models (LME) and differences between diets have been tested by analysis of variance (ANOVA). Significance was set at p < 0.1 (.), p < 0.05 (*), p < 0.01 (**) and p < 0.001 (***).

We determined fecal microbiota composition at the end of each dietary intervention, prior to acute maximal aerobic exercise test. Fecal microbiota composition was modestly affected by 1-week dietary intervention with the MedDiet or CanDiet (Fig. 5A). The order of the diets in the crossover design did not affect microbiota composition (Fig. 5B). Variables associated to the axes of the multiple factor analysis (MFA) are shown in Fig. 5C. Interindividual variability was the main factor explaining the differences between microbiota composition as hierarchical clustering of participants indicated that it had a greater impact than 1-week dietary intervention (Fig. 5D). Four bacterial families were, however, differentially modulated by the MedDiet and CanDiet. *Oscillospiraceae* and *Prevotellaceae* were significantly higher in the MedDiet compared to the CanDiet, whereas *Coriobacteriaceae* and *Erysipelotrichaceae* were significantly lower (Fig. 5E).

## Discussion and conclusions

The objective of this study was to determine if diet composition influenced the acute response post maximal exercise of circulating eCBome mediators and SCFAs in a sample of active women with no underlying metabolic conditions. Overall, our observations suggested that plasma eCBs, their congeners and some PUFAs, which are both ultimate biosynthetic precursors and metabolic products of these mediators, exhibit higher concentrations at the cessation of exercise. However, not all eCBome mediators reacted similarly, even when they belonged to the same family. The diet had a strong influence on many molecules, especially some NAEs and PUFAs, while other metabolites, including SCFAs, did not seem to be affected by the diet in the present study. Most NAEs and PUFAs increased after exercise when the participants consumed the MedDiet, but not when they ate the CanDiet. This relationship was different for 2-MAGs, which in most cases reacted similarly post-exercise with either the MedDiet or the CanDiet. Considering that fecal gut microbiota was modestly altered by the diet, the investigation of the interaction between diet, gut microbiota, eCBome and exercise were limited in this study.

This study showed that several plasmatic eCBs, eCB-like molecules and PUFAs had higher concentration at the end of aerobic exercise during MedDiet. For many of these metabolites, concentrations returned to baseline levels during the recovery phase. Previous work among heterogeneous populations globally showed that NAEs significantly increased upon 30 min of exercise of moderate intensity or over in a short-term manner^13, 14, 22, 47^. Interestingly, NAEs levels were also enhanced with a longer aerobic demand lasting up to 5 h in hypoxic conditions^15^. Some investigators have shown that AEA and OEA increased after 20 min of moderate exercise, while 2-AG, PEA and 2-OG remained stable for adult women reporting major depressive disorder^48^. The same conclusion was made among women of 27 to 43 years-of-age, where AEA and OEA increase was associated to moderate-vigorous daily exercise^49^. Therefore, it was not surprising that AEA and OEA were both responsive to aerobic exercise of maximal intensity in the present study, also considering that they share similar metabolic pathways^50^. However, Crombie and colleagues (2018) documented that not only AEA, OEA, but also 2-AG concentrations greatly increased following 30 min of moderate running in healthy adults, females in majority^51^. The increase of AEA levels after aerobic exercise could be due to the decrease of the major AEA degradation enzyme (fatty acid amide hydrolase) as shown in a middle-aged female cohort^52^. Conversely, another study in healthy people found that the long-term impact of exercise intervention was to reduce plasma AEA concentration^53^. After 30 min of arm cycling at a steady pace and incremental load, plasma levels of OEA, PEA, SEA and 2-AG were similar to levels prior to exercise after 60 min recovery, while AEA levels were slightly lower after recovery^54^, which supports the short-lasting effect of exercise on eCBome mediators. While incongruences in intervention and inclusion criteria are existent in the cited studies, these past results are coherent with the observations of the current study. However, previous studies in humans on the effect of exercise on plasma eCBs and eCB congener-like mediators did not consider the possible role of underlying dietary habits, which may also account for, or contribute to, the often slight but nevertheless important differences in the outcomes of such studies.

Indeed, we found that the diet influences the concentrations of specific eCBome mediators before and after aerobic exercise. A recent and similarly designed (but exercise-unrelated) study showed that the intake of EPA, DHA^55^, arachidonic acid (AA) and oleic acid (OA) was correlated to greater levels of plasma n-3 fatty acid- or OA-derived mediators^23^. The higher proportions of dietary and available PUFAs, MUFAs, OA, EPA, DPA and DHA in the MedDiet compared to the CanDiet could have contributed to the increase of n-3, n-6 and n-6 PUFA-derived NAE levels for participants under MedDiet. An additional explanation to the enhancer effect of the MedDiet on NAE and PUFA levels with exercise could be its modulation of muscle-specific eCB enzyme expression by both the diet^56^ and training^50^ observed in lean and obese subjects. Dietary fat intake was also associated to CB_1_ expression level reduction in the skeletal muscle in humans and rats^47, 56^ and could likewise explain the weaker alteration of eCB levels with exercise in CanDiet compared to MedDiet.

The alterations by either the diet or exercise, or both, of the plasma concentrations of eCBome mediators are likely to have functional consequences^1, 8, 57^. These molecules have several molecular targets among G protein-coupled receptors, ligand-activated ion channels and nuclear receptors, which have been implicated to different degrees in the metabolic and mood effects of food intake and exercise. In particular, although it is not possible to infer central actions from circulating endocannabinoid levels, CB_1_ receptor activation by AEA and 2-AG after the MedDiet immediately following the onset of exercise might induce anxiolytic and antidepressant action in response to stress, thus contributing to the runners “high”, and at the same time favouring food intake to replenish fuel exhausted because of exercise^8^. By contrast, in non-metabolically healthy people, CB_1_ activation in the periphery may contribute to energy accumulation and to chronic low-grade inflammation^1^. The decrease of eCBs during the process of recovery after exercise, in one or both diets, might be required to enhance the mobilization of energy necessary for physical exercise and its recovery. The elevated levels of the PPARα agonist OEA following the MedDiet and throughout the duration of exercise might allow for enhanced lipogenesis and fatty acid oxidation, again necessary for energy expenditure. For CanDiet individuals, this role might be played instead by 1/2-PG, another PPARα agonist^58^, whose levels remained higher at later times following the onset of exercise in these subjects. Stimulation by exercise of LEA and 1/2-LG levels, by activating GPR119, a glucagon-like peptide-1 releasing receptor in L cells of the intestinal epithelium, or transient receptor potential vanilloid type-1 channels in pancreatic β-cells, might contribute to counterbalancing CB_1_ activation, notably in terms of regulating insulin release and sensitivity^50, 59^. Finally, the overall increased levels of EPEA and 1/2-EPG, two potential anti-inflammatory mediators, with the MedDiet might confer to individuals under such dietary condition resistance to exercise stress-induced inflammation.

We observed a limited effect of the diet on gut microbiota composition. This was not entirely surprising as individuals who are used to carry out sport are known to harbour a more stable gut microbiota with improved metabolic functions than sedentary individuals^60^. The structural diversity and stability of the participants’ baseline gut microbiota could explain the modest impact of the diet on gut microbiota composition^61^. The effect of the dietary intervention on circulating SCFAs was also limited, as only acetic acid and isovaleric acid were significantly increased and decreased, respectively, in the MedDiet before aerobic exercise. Minimal microbiota modulation by the diets could explain why little changes in SFCA levels post exercise were observed between diets. Indeed, bacteria associated with butyrate production, for example *Lachnospiraceae* and *Ruminococcaceae*^62–64^, already had a high relative abundance in participants before intervention and their levels did not vary significantly between the diets. Nevertheless, contrasting modulation of acetate and isovalerate in response to diet and exercise might indicate that even minimal diet-induced changes in the gut microbiota of healthy lifestyle young healthy individuals may have functional importance, as acetate is considered beneficial and isovalerate detrimental for metabolic health^65^. In past studies, SCFA levels in feces were related to diet and an active lifestyle^66–70^. Previous studies mostly assessed fecal concentrations after exercise^60, 63^, or in cohorts of athletes compared to controls^71^. Additional studies suggested that propionic acid and butyric acid increased with exercise^72–75^. Our findings provide new insight on SCFA modulation by maximal exercise.

In summary, this study investigated the response to acute maximal aerobic test of an extensive array of eCBome mediators, covering NAEs, MAGs, and some of their corresponding free fatty acids. It also provided profiles for acute SCFA levels in plasma following exercise. A clear limitation of our study is the small size of the cohort sampled. However, we overcame this limitation by using a randomized crossover design, which allowed participants to act as their own controls. Additionally, we submitted the participants to stringent admissibility criteria and to a controlled full-feeding program prior to the acute maximal aerobic test, which minimized many transient and permanent confounding variables that could have affected the results. These strategies helped to compensate for the potential problems arising from the use of a small sample of participants and allowed to observe statistically significant alterations by either diet, exercise, or both. An important strength of the study is the selection of a homogenous population characterized by women living an active lifestyle. This cohort enabled to distinguish the effect of intense physical activity from extreme homeostatic stress, which unfit individuals would have faced. Overall, our study shows that eCBome mediators react to acute exercise in a way that is dependent on the diet consumed in the week prior to exercise.

## Data Availability

Raw sequencing reads are available in SRA (PRJNA758983).
